# Knockdown of DJ-1 Exacerbates Neuron Apoptosis Induced by TgCtwh3 through the NF-κB Pathway

**DOI:** 10.1007/s12035-024-04265-7

**Published:** 2024-06-03

**Authors:** Di Yang, Minmin Wu, Nian Zou, Yiru Tang, Qing Tao, Lei Liu, Mengmeng Jin, Li Yu, Jian Du, Qingli Luo, Jilong Shen, Deyong Chu, Kunpeng Qin

**Affiliations:** 1 Department of Pathogen Biology, Anhui Province Key Laboratory of Microbiology & Parasitology, Anhui Provincial Laboratory of Zoonoses of High Institutions, School of Basic Medicine, Anhui Medical University, Hefei, China; 2 Department of Orthopaedics, The First Affiliated Hospital of Anhui Medical University, Hefei, China; 3 Department of Orthopaedics, Anhui Public Health Clinical Center, Hefei, Anhui China; 4 Department of Clinical Laboratory, The First Affiliated Hospital of Anhui Medical University, Hefei, China; 5 Second School of Clinical Medicine, Anhui Medical University, Hefei, China; 6 School of Public Health, Anhui Medical University, Hefei, China; 7 Center for Translational Medicine, Jiangsu Key Laboratory of Molecular Medicine, Medical School of Nanjing University, Nanjing, China; 8 Department of Blood Transfusion, Division of Life Sciences and Medicine, the First Affiliated Hospital of USTC, University of Science and Technology of China, Hefei, China; 9 Maternity and Child Health Hospital of Anhui Province, the Affiliated Maternity and Child Health Hospital of Anhui Medical University, Hefei, China; 10 Department of Microbiology and Parasitology, Anhui Provincial Laboratory of Microbiology and Parasitology, Anhui Provincial Laboratory of Zoonoses of High Institutions, School of Basic Medical Sciences, Anhui Medical University, Hefei, China; 11 Department of Biochemistry and Molecular Biology, School of Basic Medical Sciences, Anhui Medical University, Hefei, China

**Keywords:** DJ-1, TgCtwh3, Apoptosis, NF-κB, Hippocampal neurons

## Abstract

Mutations or loss of function of DJ-1 and *Toxoplasma gondii* (*T. gondii*) infection has been linked to neurodegenerative diseases, which are often caused by oxidative stress. However, the relationship between DJ-1 and *T. gondii* infection is not yet fully understood. Therefore, this study aimed to investigate the expression of DJ-1 in the hippocampus tissue of mice or in HT22 infected with *T. gondii* Chinese 1 genotype Wh3 strain (TgCtwh3) and the effect of DJ-1 knockdown on neuronal apoptosis induced by TgCtwh3 tachyzoite, as well as the underlying mechanism at the cellular and molecular level. Firstly, we detected DJ-1 protein expression and cell apoptosis in the hippocampal tissue of mice infected by TgCtwh3. Then, we examined DJ-1 expression and apoptosis in HT22 challenged with TgCtwh3. Finally, we evaluated the apoptosis in HT22 with DJ-1 knockdown which was infected with TgCtwh3 and assayed the expression of NF-κBp65 and *p*-NF-κBp65. Our results showed that DJ-1 expression was reduced and neurons underwent apoptosis in the hippocampus of mice infected with TgCtwh3 tachyzoites. Additionally, the knockdown of DJ-1 followed by infection with TgCtwh3 tachyzoites led to increased apoptosis in HT22 cells through the NF-κB signaling pathway. Therefore, this study suggests that DJ-1 is an important target for preventing apoptosis caused by *T. gondii* TgCtwh3.

## Introduction

*Toxoplasma gondii* (*T. gondii*) is a zoonotic intracellular parasitic protozoan that has infected approximately 30% of the global human population [1]. The transmission of *T. gondii* in populations is primarily associated with hygiene and dietary habits, as infection occurs mainly through the ingestion of water and food contaminated with encapsulated toxoplasmosis [1, 2]. This is due to differences in geographic regions and living environments [3]. *T. gondii* is neurophilic and can cause damage to immunodeficient individuals, such as AIDS patients and organ transplant recipients [4]. It is important to note that *T. gondii* infects individuals, usually forming cysts in the brain, muscles, and liver [5], but tends to infect the central nervous system, causing brain infections and encephalitis, among other conditions [6]. Prolonged latent infection can lead to a variety of psychiatric disorders, including Parkinson’s disease, Alzheimer’s disease, and schizophrenia [7]. Some studies indicate that *T. gondii* has the ability to breach the blood-brain barrier and invade neurons through three mechanisms: paracellular entry [8], transcellular migration [5], and infiltration of infected immune cells (known as the “Trojan horse” mechanism) [7, 9, 10]. This enables the parasite to access the central nervous system and infect neurons, which play a crucial role in the central nervous system (CNS) infection.

DJ-1/PARK7 is a homodimeric protein that is highly conserved. Its deletion or mutation in the gene encoding is closely associated with autosomal recessive early-onset Parkinson’s disease [11, 12]. The amino acid sequence of DJ-1 contains three cysteine residues at positions 46, 53, and 106 [13]. The function of DJ-1 is determined by the oxidation and nitrosylation of these cysteine residues [14, 15]. Although initially identified as an oncogene, DJ-1 has been found to exert cytoprotective effects through resistance to oxidative stress [16]. The neuroprotective function of DJ-1 is mainly attributed to its effects on mitochondrial maintenance and antioxidant properties [17]. It has been observed that increased oxidative stress leads to downregulation of the protective factor DJ-1, which is closely related to the level of oxidative stress in AD [18]. Pharmacological studies have demonstrated that DJ-1 is essential for the protective effect of specific compounds that are known to hinder the production of reactive oxygen species. This hindrance prevents oxidative stress-induced cell death in neuroblastoma, dopaminergic cells, and primary neuronal cells when these compounds are present [19]. The overexpression of DJ-1 resulted in a decrease in BAX expression and inhibited caspase activation. Conversely, the knockdown of DJ-1 increased BAX protein levels, caspase-3 activation, and cell death induced by UV irradiation [20, 21]. Furthermore, it was discovered that DJ-1 interacts directly with p53 and its sumoylated form inhibits p53’s transcriptional activity [22].

Numerous studies have indicated that DJ-1 is linked to neurological disorders such as Parkinson’s disease, Alzheimer’s disease, and depression. Furthermore, DJ-1 is involved in various physiological processes, including anti-oxidative stress [23] and anti-apoptosis [24]. It has been demonstrated by research and meta-analysis data that there could be a relationship between toxoplasmosis and the onset of dementia including Alzheimer’s disease (AD) [25–27]. As a result, few studies have investigated the impact of DJ-1 and *T. gondii* infection on neurons. The Chinese 1 genotype (ToxoDB #9) of *T. gondii*, which is prevalent in China, was discovered by our research team [28]. Our study has identified two representative strains, namely *T. gondii* Chinese 1 genotype Wh3 strain (TgCtwh3) and *T. gondii* Chinese 1 genotype Wh6 strain (TgCtwh6), both of which are associated with this genotype and belong to the Chinese 1 genotype of *T. gondii* [28]. Therefore, we investigated the alterations in DJ-1 and apoptosis indexes in mouse hippocampal neurons following *T. gondii* infection. Additionally, we examined the changes in neuronal apoptosis by knocking down DJ-1 in neuronal cells in vitro and subsequently infecting them with *T. gondii* tachyzoites.

## Materials and Methods

### Antibodies and Materials

Anti-DJ-1 antibodies were purchased from Abcam (Cambridge, US). Anti-p53, Bax, and cleaved caspase-3 antibodies were purchased from Wanleibio (Shenyang, China). FITC-labeled goat anti-rabbit IgG, phenylmethanesulfonyl fluoride (PMSF), and phosphorylated protease inhibitors were purchased from Servicebio (Wuhan, China). Anti-GAPDH and goat anti-rabbit antibodies were purchased from Proteintech (Chicago, IL, USA).

Dulbecco’s modified Eagle’s medium (DMEM) and fetal bovine serum (FBS) were obtained from Biological Industries (Palestine). 2-(4-Amidinophenyl)-6-indolecarbamidine dihydrochloride (DAPI), penicillin-streptomycin, Annexin V-FITC Apoptosis Detection kit, and SDS polyacrylamide gel electrophoresis were purchased from Beyotime (Shanghai, China). Hematoxylin and eosin (H&E) and Nissl staining kit were purchased from Sigma (St. Louis, MO, USA). BCA protein assay kits was obtained from Biosharp (Hefei, China). RIPA lysis buffer and nitrocellulose membrane were provided by Millipore (Billerica, MA, USA). The hippocampal neuronal cell line (HT22) and Vero cell line (Vero) were purchased from Procell Life Science (Wuhan, China). TgCtwh3 tachyzoites were kept in Vero cells, respectively, which were stored at − 80℃ in our laboratory (Anhui Province Key Laboratory of Microbiology and Parasitology).

### Animals

Twenty female BABL/c mice, aged 8 to 10 weeks, were acquired from Hangzhou Ziyuan Experimental Animal Technology Company in Zhejiang, China (production permission number: Scxk 2019-0004). The mice were specified pathogen free (SPF) and averaged 18 to 20 g. The mice were handled in strict accordance with the Chinese National Institute of Health Guide for the Care and Use of Laboratory Animals. The Institute of Biomedicine at Anhui Medical University’s Institutional Review Board approved this animal experiment (permission number: AMU26093628). All mice were housed in the colony room of the Anhui Province Key Laboratory of Microbiology and Parasitology under controlled conditions (12/12-h circadian rhythm, temperature of 20 ± 2 °C, humidity of 45 ± 5%), and were provided normal chow and pure water ad libitum.

Following a week of eating, all mice were randomly separated into two groups of ten: control group and TgCtwh3 group. Each mouse in the TgCtwh3 group received an intraperitoneal injection of 1000 TgCtwh3 tachyzoites in 200 µl normal saline, whereas each mouse in the control group received an intraperitoneal injection of 200 µl normal saline. On the seventh day after infection, all mice were put to death, and their brain tissues were collected for histological examination and the detection of protein and gene expression.

### Plasmids and siRNA

The present study reports the construction of a plasmid for human DJ-1 expression [29]. The open reading frame that encodes DJ-1 was amplified from human RNA (GenBank ID: GU175984.1) and subcloned into the pSin-HA vector. Mouse hippocampal neuronal cell lines were transfected with anti-DJ-1 siRNA, which was obtained from RiboBio Co, Ltd (Guangzhou, China), following the manufacturer’s instructions. The DJ-1 siRNA used in this study was DJ-1 siRNA #: GGTCATTACACCTACTCTG (sense). The cells were inoculated in 12-well plates containing antibiotic-free medium and cultured to a density of 30–50% for 24 h before transfection.

### Cell Culture

HT22 cells and Vero cells were respectively cultured in DMEM containing 10% FBS, 1% penicillin-streptomycin in an incubator at 37 °C, and 5% CO_2_. HT22 cells were infected with TgCtwh3 tachyzoites, HT22 cells were knocked down with siRNA, and groupings were set up as control, si-NC, si-DJ-1, TgCtwh3, si-NC + TgCtwh3, and si-DJ-1 + TgCtwh3 for the subsequent experiments.

### Western Blot

RIPA lysis buffer combined with protease inhibitor was used to lyse approximately 80 mg of hippocampus tissue or cultivated HT22 cell line. A BCA protein concentration assay kit was used to assess the extracted total protein content. Each protein sample (10 µg) was put into a 12.5% polyacrylamide gel and then transferred to a polyvinylidene difluoride membrane (PVDF) for electrophoresis and separation. Protein-free fast-blocking solution was used to prevent non-specific binding for roughly 20 min at room temperature. DJ-1 (1:5000), caspase-3 (1:1000), p53 (1:2000), and GAPDH (1:5000) were treated with membranes. The secondary antibodies (1:10,000) were then treated with the primary antibodies at room temperature for 1 h after being incubated at 4 °C for an overnight period. The ECL chemiluminescence kit was used to find specific protein signals. The blot pictures were viewed using the Chemo Dox XRS system (Bio-Rad Laboratories, Hercules, CA, USA). The Image J program (MD, USA) was used to calculate the optical density of each band.

### Hematoxylin and Eosin (HE) Staining

At least three mice from each group were randomly selected, and 4-mm paraffin slices of their brains were dewaxed three times in xylene before being hydrated in ethanol at a gradient concentration. Dewaxed paraffin sections were soaked in hematoxylin for 5 min before being rinsed slowly with running water for several seconds. They were then differentiated with 1% hydrochloric acid alcohol for 30 s before being rinsed slowly with running water for several seconds. These parts were then submerged in eosin for 25 s to 1 min. Finally, the sections were systematically dehydrated in anhydrous ethanol, permeabilized in xylene, sealed with neutral glue, and allowed to dry naturally. Under a light microscope (LEICA, Wetzlar, Germany) with magnifications of 20 × 10 and 40 × 10, all HE-stained tissue sections were examined, and the pictures in the field of vision of the hippocampus zone for each section were recorded by an observer in a blinded manner.

### Nissl Staining

Nissl staining was used to observe changes in the number of intra-neuronal nissl bodies in the hippocampus of *T. gondii*-infected mice. Dewaxed paraffin slices were submerged in a tarry violet staining solution and 56 °C for 1 h. It was cleaned slowly with deionized water. The portions were then submerged for 2 min in nissl differentiation solution. Under a microscope, note the degree of distinction. After that, xylene was used to make the dehydration translucent before the neutral resin was used to close the process. To conduct the final Image J (USA) analysis, all sections were examined under an optical microscope (40 × 10) (LEICA, Wetzlar, Germany) and the total number of positive regions was counted.

### Immunohistochemistry (IHC)

After brain tissue sections (both uninfected and TgCtwh3-infected groups) were deparaffinized three times in xylene, they were rehydrated in graded ethanol concentrations and then subjected to antigen repair in sodium citrate solution. After that, an appropriate amount of endogenous peroxidase blocker was added dropwise and incubated at room temperature for 10 min. Finally, the sections were incubated with anti-DJ-1 (1:500) overnight at 4 °C and then with HRP-coupled goat anti-rabbit IgG for 30 min at 37 °C. Chromatography was carried out with DAB, and nuclei were stained with hematoxylin. All tissue sections were observed under a light microscope at 20 × 10 and 40 × 10 magnifications, and images of randomly different sites of each section were captured by blinding. Quantitative and qualitative changes were also analyzed using morphometric software.

### Immunofluorescence Staining

TgCtwh3 tachyzoites were harvested from continuous cultures in Vero. HT22 was divided into groups of two or six and cultured in 12-well plates with coverslips in each well. After HT22 was cultured, at various periods, the cells in the corresponding wells were transiently transfected, as well as TgCtwh3 tachyzoites (Multiplicity of infection, MOI = 3) were added, and then, the culture was continued. Finally, HT22 was then fixed with 4% paraformaldehyde after being washed with PBS. After 0.5% Triton X-100 was used to penetrate cell membranes, the cells were incubated with anti-DJ-1 antibody (1:500) for 12 h, respectively. Following this, the cells were incubated with FITC-coupled goat anti-rabbit antibody (1:200) for 1 h at 37 °C, and the nuclei of the cells were then again stained with DAPI. After applying an anti-fluorescence quencher, coverslips were examined with a 40 × 10 fluorescent microscope. For each crawl sheet, the observer took pictures in five randomly selected fields of view.

### Flow Cytometry (FCM)

After the process of tachyzoite infection and siRNA transfection and re-infection of HT22 cells, the cell suspension was extracted, and the apoptosis of HT22 cells was detected by FITC Annexin V Apoptosis Detection Kit and CytoFLEX flow cytometry. Finally, the results were analyzed by FlowJo_V10 for data analysis.

### Statistical Analysis

All data were obtained from triplicate values representing three independent experiments with identical conditions. One-way ANOVA followed by the Bonferroni post hoc test was used for data analysis using GraphPad Prism 8.2.1 (GraphPad Software, San Diego, CA, USA). All results were assessed as mean ± SD (*n* = 5 replicates for each group), and two-tailed *P* < 0.05 or *P* < 0.01 or *P* < 0.001 was regarded as statistically significant.

## Results

### Reduced Expression of DJ-1 in the Hippocampal Region of the Brain of TgCtwh3-Infected Mice

**Fig. 1:**
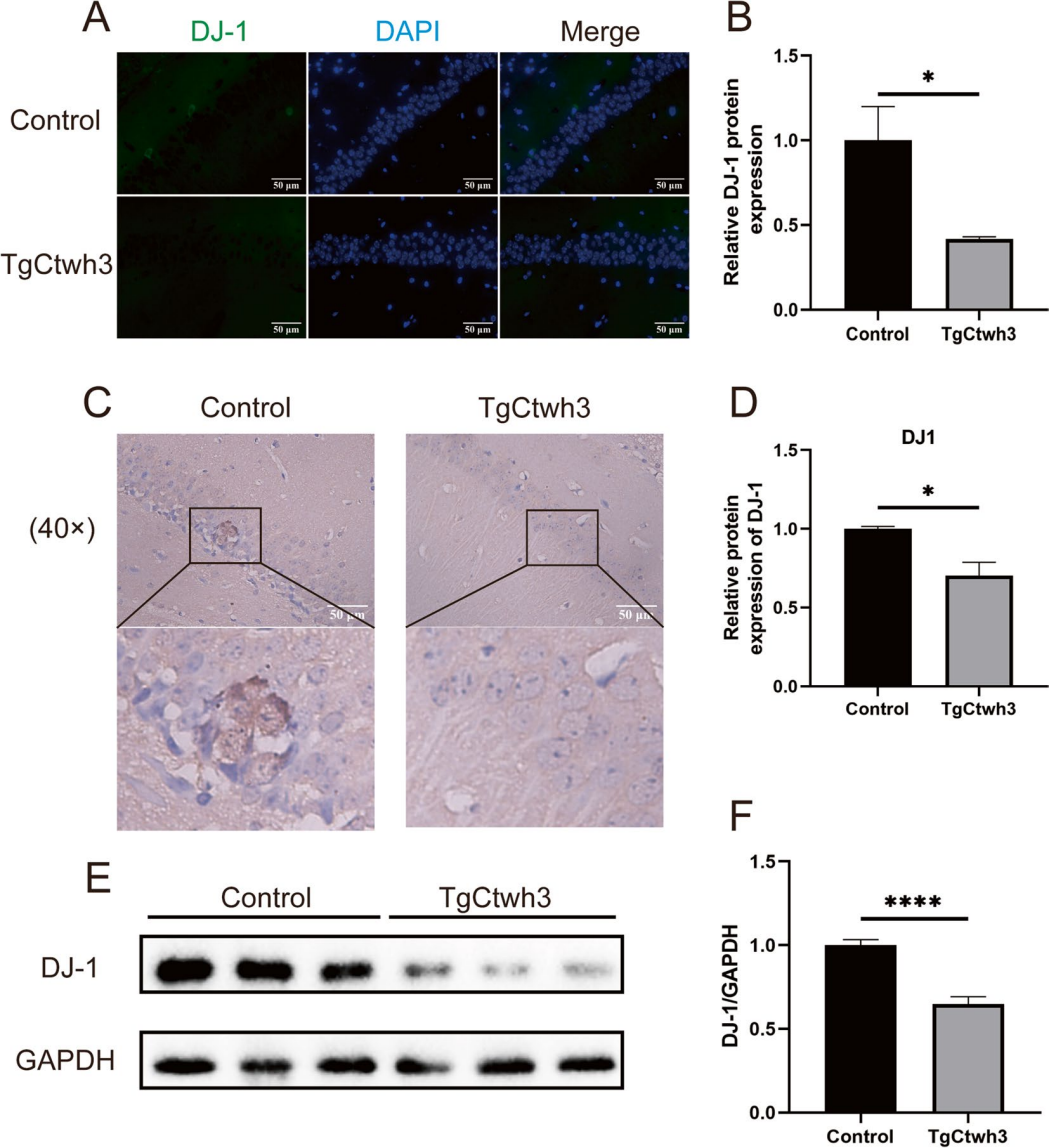
Decreased DJ-1 expression occurs in the hippocampus of TgCtwh3-infected mice. DJ-1 protein expression was observed in mouse hippocampal tissue through immunofluorescence staining and viewed under a fluorescence microscope at a magnification of 40 × 100 (scale bar, 50 μm) (**A**, **B**). The hippocampal tissue sections were analyzed through immunohistochemistry (scale bar, 50 μm) (**C**, **D**). Western blotting was conducted to detect DJ-1 protein expression in mouse hippocampal tissue (**E**, **F**). The data, presented as mean ± SEM, were analyzed by one-way ANOVA followed by Bonferroni post hoc test for multiple comparisons (*n* = 3 per group). *P < 0. 05, **P < 0.01, ***P < 0.001

To investigate the expression of DJ-1 in the hippocampal region of *T. gondii*-infected mice, we conducted a histological examination of the infected mice’s brains. Our findings revealed that DJ-1 expression was reduced in the hippocampal tissue of TgCtwh3-infected mice compared to the control group, as evidenced by the results of immunofluorescence staining (Fig. 1A, B). Furthermore, the results of immunohistochemistry indicated a significant reduction in DJ-1 protein expression in the hippocampal region of TgCtwh3-infected mice compared to the control group (Fig. 1C, D). Finally, Western blotting results showed a significant decrease in the level of DJ-1 protein expression in the hippocampal tissues of mice in the TgCtwh3-infected group compared to the control group (Fig. 1E, F). In conclusion, our findings suggest that DJ-1 expression is reduced in the hippocampal region of the brain in mice infected with *T. gondii*.

### TgCtwh3 Infection Causes Histopathological Changes in the Hippocampal Region of Mice

**Fig. 2:**
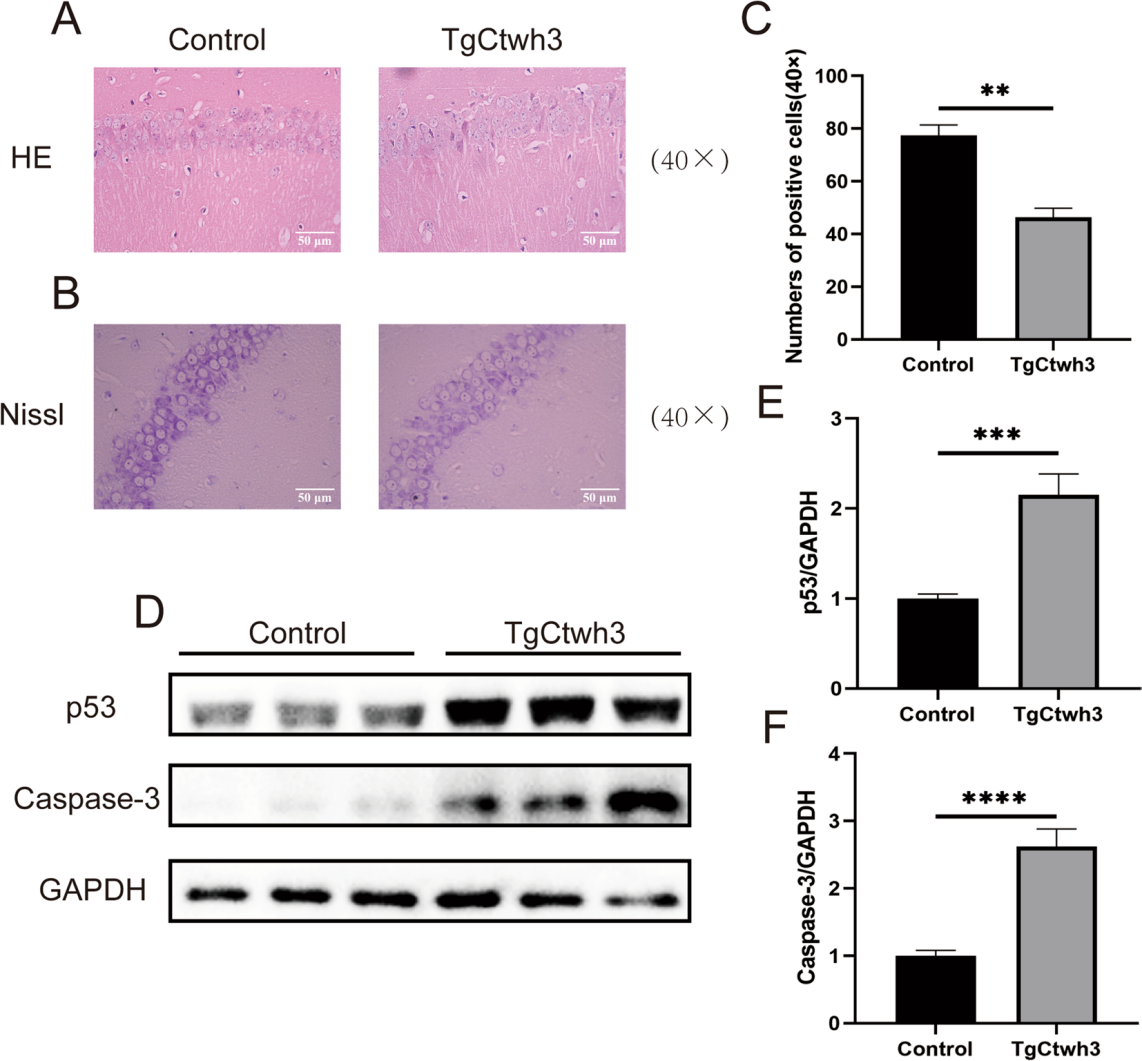
TgCtwh3 infection leads to the apoptosis of hippocampus cells in mouse. HE-stained mouse hippocampal tissue sections (scale bar, 50 μm) of the control group were compared with the infected group (**A**). Mouse hippocampal tissue sections were stained with Nissl dye, and then, neurons were observed semi-quantitatively under a light microscope at a magnification of 40 × 100 (scale bar, 50 μm) (**B**, **C**). Western blotting was performed to detect the expression of apoptosis-related proteins, P53 and caspase-3, in the hippocampal tissues of mice (**D**–**F**). The data, presented as mean ± SEM, were analyzed by one-way ANOVA followed by Bonferroni post hoc test for multiple comparisons (*n* = 3 per group). *P < 0. 05, **P < 0.01, ***P < 0.001

To explore whether TgCtwh3 infection induces pathological alterations in the brain tissue of the hippocampal region of mice, a histological examination of the hippocampal region was conducted. The results of HE staining revealed that the hippocampal tissues of infected mice exhibited structural incompleteness, reduced neuronal count, disorganized cell arrangement, decreased cell volume, irregular and deeply stained nuclei, and unclear nuclear membrane boundaries, in contrast to the control group mice (Fig. 2A). Furthermore, the results of Nissl staining demonstrated a significant decrease in the number of Nissl bodies in the hippocampal neurons of the infected group, indicating a reduction in the number of hippocampal nerves due to TgCtwh3 infection (Fig. 2B, C). Western blotting analysis revealed an upregulation of apoptotic proteins, p53, and caspase-3, in hippocampal neurons of the infected group compared to the control group (Fig. 2D–F). Collectively, these findings suggest that TgCtwh3 infection induces alterations in the number, morphology, and arrangement of mouse hippocampal neurons, leading to apoptosis.

### Direct Infection on HT22 Cells by TgCtwh3 Downregulates DJ-1 Expression

**Fig. 3:**
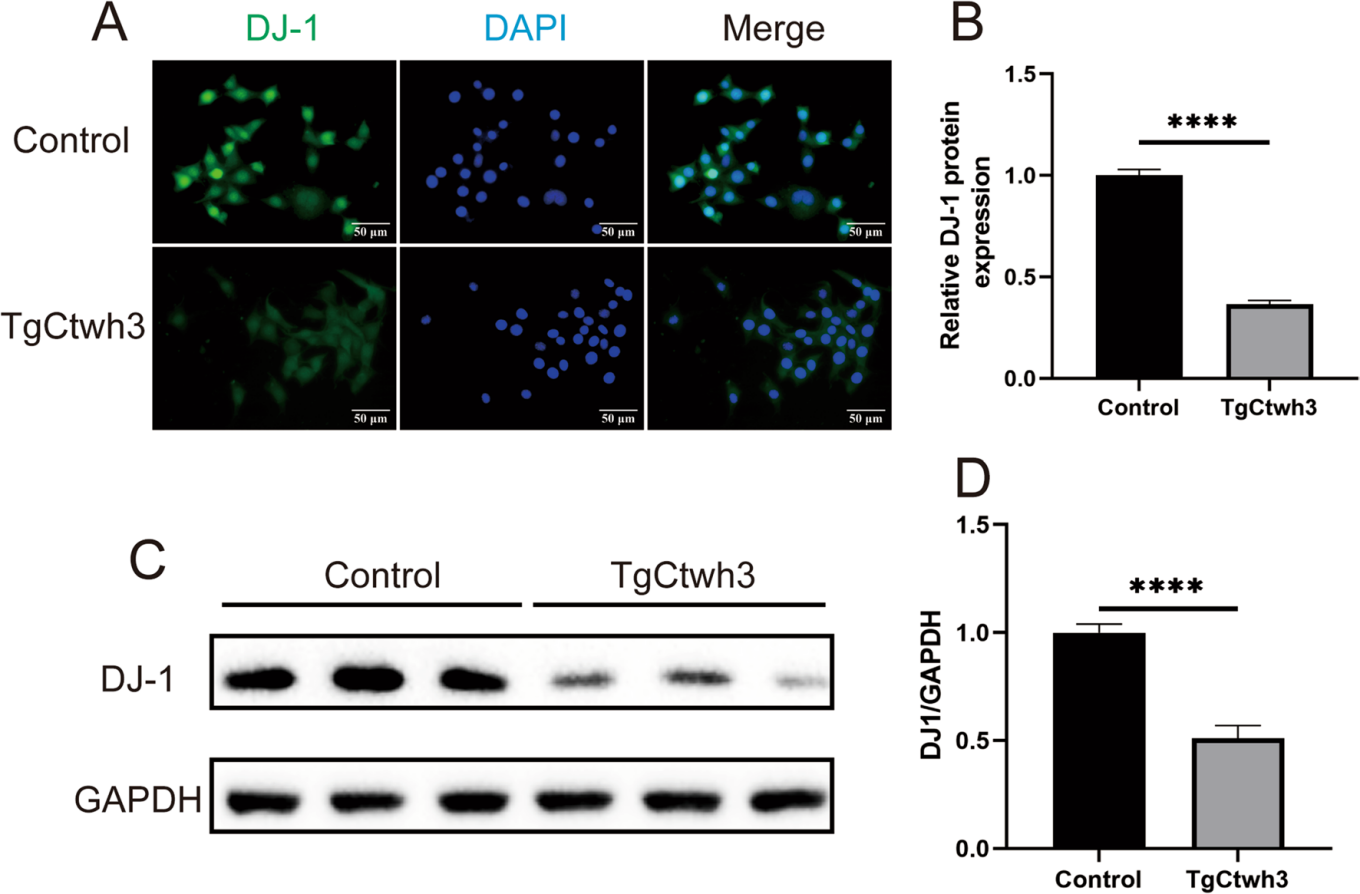
TgCtwh3 tachyzoites downregulate DJ-1 expression in HT22. Immunofluorescence staining was performed on HT22 cells to observe the expression of DJ-1 after infection with TgCtwh3 tachyzoites (**A**, **B**). The expression of DJ-1 in HT22 cells was then detected by Western blotting after staining with TgCtwh3 tachyzoites (**C**, **D**). The data, presented as mean ± SEM, were analyzed by one-way ANOVA followed by Bonferroni post hoc test for multiple comparisons (*n* = 3 per group). *P < 0. 05, **P < 0.01, ***P < 0.001

After incubating HT22 cells in vitro with TgCtwh3 tachyzoites for 24 h, a significant decrease in DJ-1 expression was observed through immunofluorescence staining (Fig. 3A, B). Furthermore, changes in DJ-1 expression were detected in HT22 cells via Western blotting, revealing a significant reduction in the level of DJ-1 protein in the infected group of cells (Fig. 3C, D). This finding indicates that infection with TgCtwh3 tachyzoites results in the suppression of DJ-1 expression in the hippocampal neurons of mice.

### TgCtwh3 Infection on HT22 Causes Direct Apoptosis

**Fig. 4:**
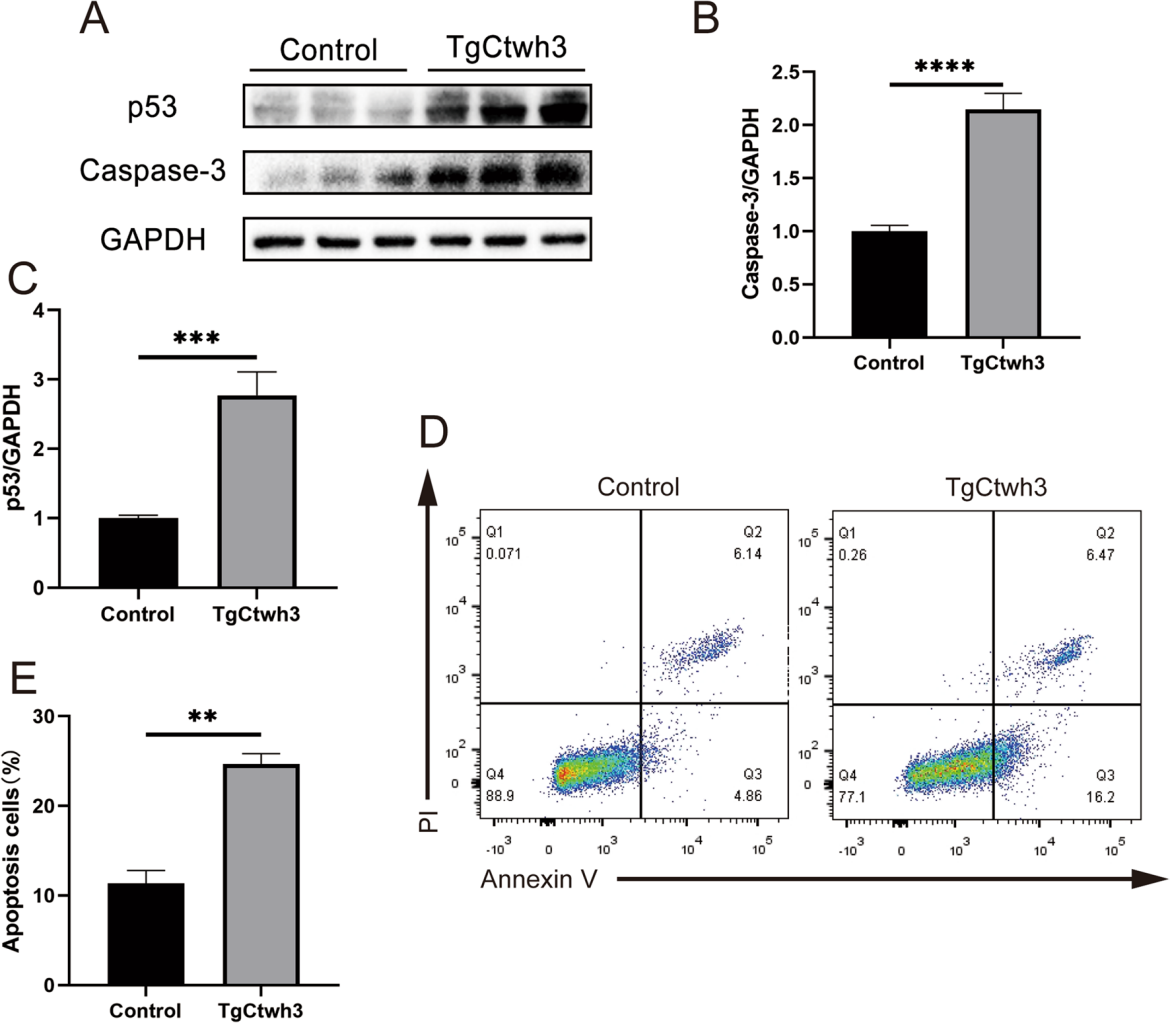
TgCtwh3 tachyzoites directly induce apoptosis in HT22 cells. Western blotting was performed to detect the expression of apoptosis-related proteins P53 and caspase-3 in HT22 cells infected with TgCtwh3 tachyzoites (**A**, **B**, **C**). FCM was performed to detect apoptosis in HT22 cells (**D**, **E**). The data, presented as mean ± SEM, were analyzed by one-way ANOVA followed by Bonferroni post hoc test for multiple comparisons (*n* = 3 per group). *P < 0. 05, **P < 0.01, ***P < 0.001

Our in vivo experiments showed that TgCtwh3 infection resulted in hippocampal cell apoptosis and hippocampal neuronal number loss. In order to elucidate whether TgCtwh3 could give rise to neuronal apoptosis, we conducted Western blotting to detect the expression of apoptosis-related proteins, p53 and caspase-3, in TgCtwh3-infected HT22 cells. The results indicated a significant upregulation in the expression of p53 and caspase-3 proteins (Fig. 4A–C). Additionally, flow cytometry analysis demonstrated a significant increase in the rate of early and late apoptosis in HT22 cells in the infected group compared to the control group (Fig. 4D, E). Therefore, it can be concluded that TgCtwh3 infection directly induces neuronal apoptosis in the hippocampal region of infected mice.

### Knockdown of DJ-1 Expression in HT22 Cells

**Fig. 5:**
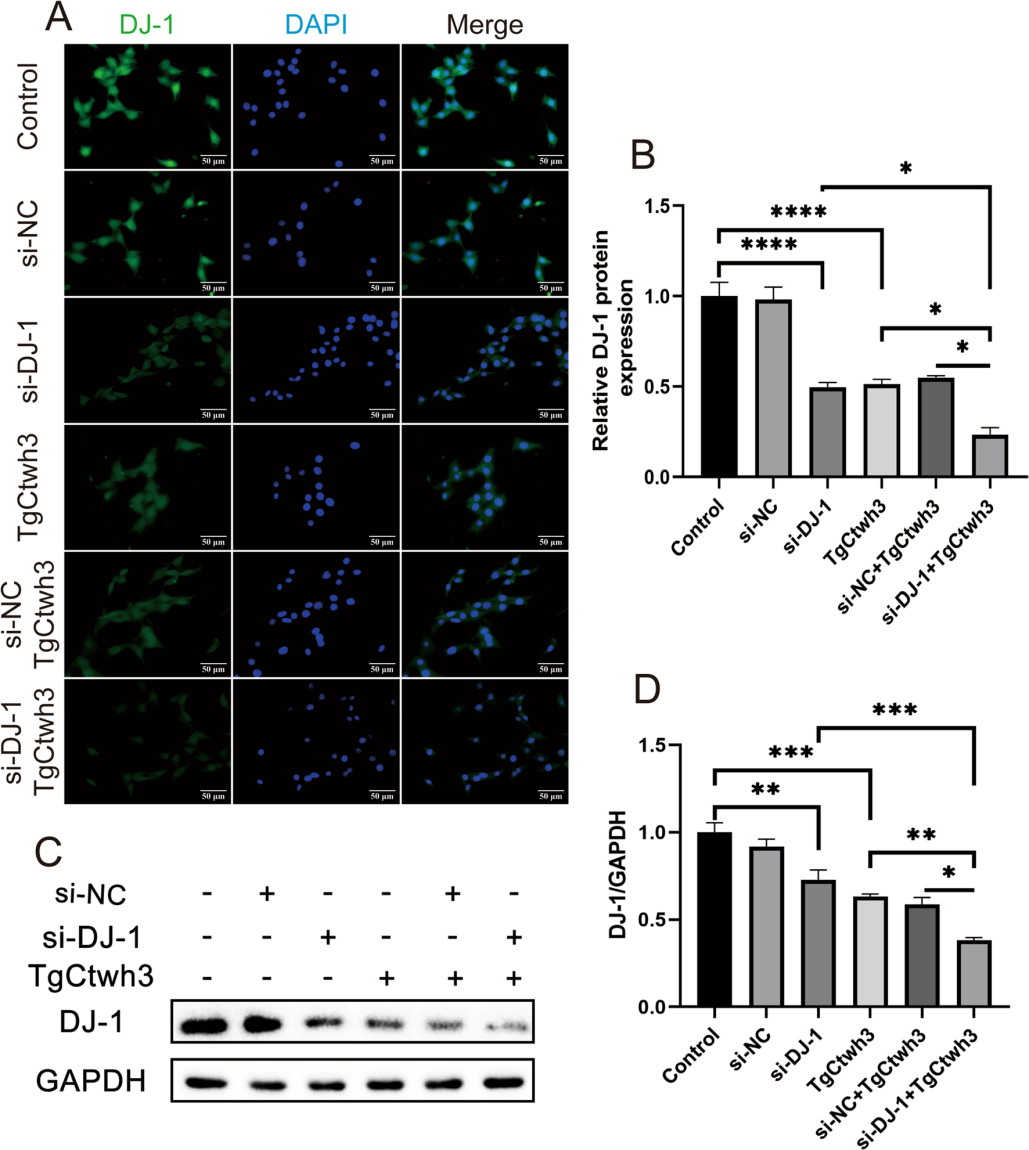
HT22 cells knock down DJ-1 expression. The DJ-1 gene was knocked down by siRNA transfection of HT22 cells and infected with TgCtwh3 tachyzoites, and each group of DJ-1 was firstly stained and observed by immunofluorescent dye (**A**, **B**). Then, the expression of DJ-1 protein in each group was detected by Western blotting (**C**, **D**). The data, presented as mean ± SEM, were analyzed by one-way ANOVA followed by Bonferroni post hoc test for multiple comparisons (*n* = 3 per group). *P < 0. 05, **P < 0.01, ***P < 0.001

To confirm the effectiveness of siRNA-mediated knockdown of DJ-1, we initially assessed DJ-1 expression following *T. gondii* infection of HT22 cells. Results from both immunofluorescence staining (Fig. 5A, B) and Western blotting (Fig. 5C, D) demonstrated a significant reduction in DJ-1 protein expression in the si-DJ-1 group, TgCtwh3 group, and si-NC + TgCtwh3 group, as compared to the normal group and si-NC group (transfected with an empty vector). Furthermore, DJ-1 expression was weakest in the TgCtwh3 + si-DJ-1 group. Notably, there was no statistically significant difference observed between the normal and si-NC groups, nor between the TgCtwh3 and TgCtwh3 + si-DJ-1 groups.

### Knockdown of DJ-1 Expression in HT22 Cells Followed by Infection with TgCtwh3 Tachyzoites Can Lead to Aggravated Apoptosis via the NF-κB Pathway

**Fig. 6:**
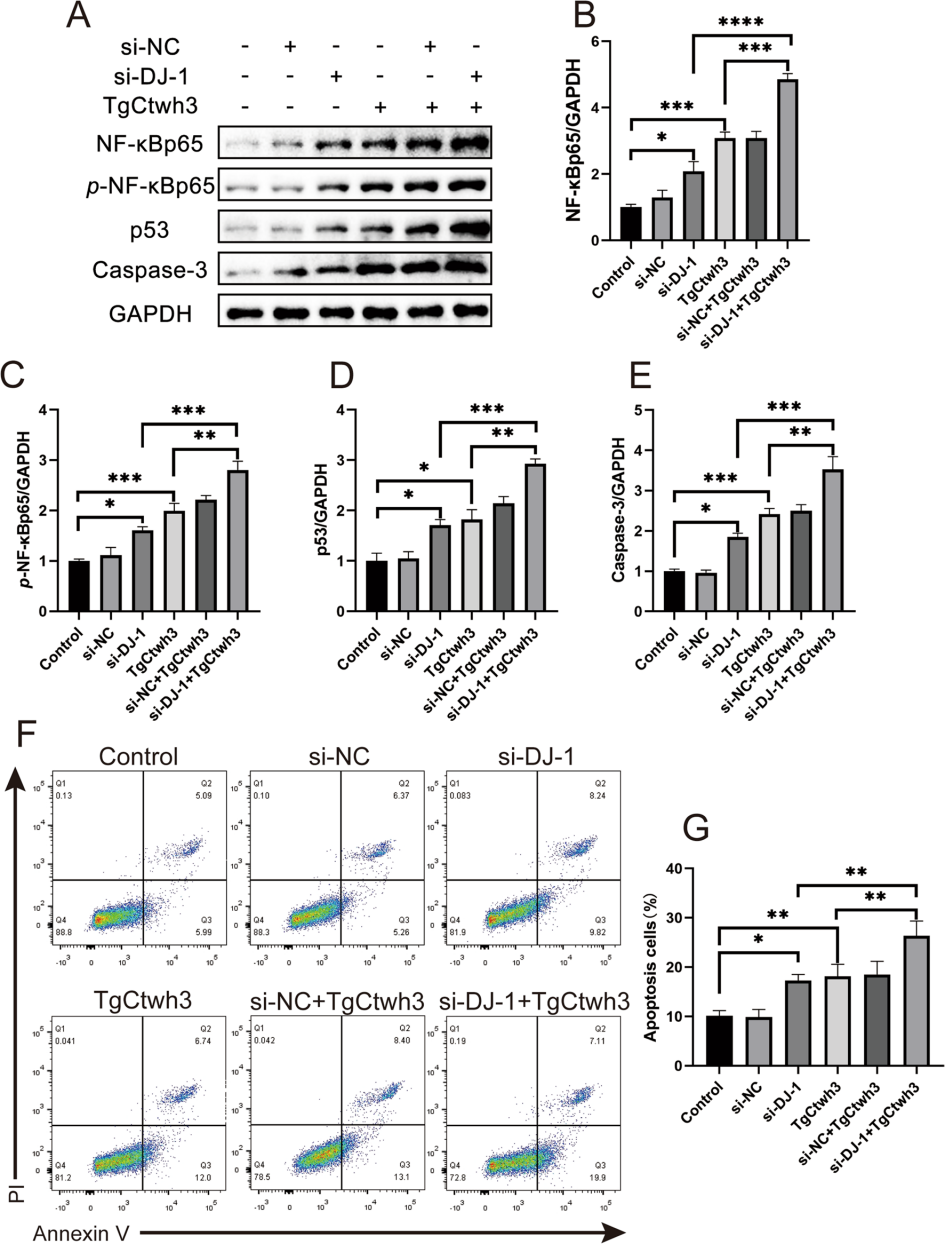
Knocking down DJ-1 expression in HT22 cells and then infecting them with TgCtwh3 tachyzoites caused increased apoptosis through the NF-κB pathway. Western blotting detected NF-κBp65, *p*-NF-κBp65, P53, and caspase-3 expression in HT22 cells, followed by semi-quantitative analysis (**A**–**E**). The apoptosis rate of HT22 cells in each group was further detected by FCM (**F**, **G**). The data, presented as mean ± SEM, were analyzed by one-way ANOVA followed by Bonferroni post hoc test for multiple comparisons (*n* = 3 per group). *P < 0. 05, **P < 0.01, ***P < 0.001

According to previous research, DJ-1 functions as a sensor for oxidative stress [30], counteracting apoptosis by promoting nuclear translocation of NF-κB to inhibit ROS production and regulate mitochondrial function [31]. Additionally, GRA15_II_ is a dense granule protein of *T. gondii* that is required for p65 nuclear translocation and NF-κB-mediated transcription in host cells [32, 33]. The NF-κB pathway plays a crucial role in the host’s innate and adaptive immune system [34]. Our objective was to investigate the apoptosis of HT22 cells after knocking down DJ-1 and infecting them with TgCtwh3 tachyzoites, as well as whether the NF-κB signaling pathway is involved in HT22 apoptosis. Western blotting revealed that the expression of NF-κBp65, *p*-NF-κBp65, and apoptosis-related proteins p53 and caspase-3 proteins were significantly elevated in the TgCtwh3 group, the si-DJ-1 group, and the TgCtwh3 + si-NC group compared with the control and si-NC groups (Fig. 6A–E). The TgCtwh3 + si-DJ-1 group had the highest expression of NF-κBp65, *p*-NF-κBp65, p53, and caspase-3 proteins. Furthermore, flow results showed that the early and late apoptosis rates were significantly higher in the TgCtwh3 group, si-DJ-1 group, and TgCtwh3 + si-NC group compared with the control and si-NC group, with the highest apoptosis rate observed in the TgCtwh3 + si-DJ-1 group (Fig. 6F, G). Therefore, our findings suggest that knocking down DJ-1 and infecting with TgCtwh3 tachyzoites had a promoting effect on apoptosis in mouse hippocampal neurons, which may be due to the activation of the NF-κB signaling pathway in neurons.

## Discussion

Infection with *T. gondii* has been observed to cause cell death in various types of cells, including neurons [35]. DJ-1, a protein with antioxidant properties, has been found to protect against cell death by translocating into mitochondria [36]. The findings of our study indicate that *T. gondii* TgCtwh3 tachyzoites directly induce apoptosis in mouse hippocampal neurons and result in a reduction of DJ-1 expression. Furthermore, our research also demonstrates that knocking down DJ-1 expression in HT22 cells leads to an increase in apoptosis following infection with TgCtwh3 tachyzoites.

The DJ-1 gene was initially discovered as a new mitogen-dependent oncogene that participates in the Ras-dependent signal transduction pathway [37]. The DJ-1 protein possesses several specific mechanisms that safeguard dopaminergic neurons against neurodegeneration in Parkinson’s disease [38–40]. It provides protection to dopaminergic neurons from oxidative damage both in vitro and in vivo [15, 41]. Oxidized DJ-1 has been observed to be markedly decreased in the brains of individuals with idiopathic Parkinson’s disease, indicating that DJ-1-mediated composite functional changes may also contribute to the pathogenesis of the more prevalent sporadic form of the disease [42]. *T. gondii* infection is known to be associated with neurodegenerative diseases [43], and from our results, we found that TgCtwh3 tachyzoite infection resulted in decreased DJ-1. And in in vitro experiments, DJ-1 was knocked down in HT22 cells, and DJ-1 expression was even lower when re-infected with TgCtwh3 tachyzoites.

It is known that an effective antioxidant system is crucial for the establishment and survival of *Toxoplasma* infection [44]. Additionally, *T. gondii* is capable of infecting various host cell types due to its resistance to externally applied peroxides [45]. DJ-1 is considered an oxidative stress-responsive protein [46] that plays a significant role in regulating oxidative stress in neuronal cells [47]. Therefore, reduced levels of DJ-1 due to *T. gondii* infection can lead to the development of oxidative stress in the mouse brain. Oxidative stress is a significant pathological manifestation of neurodegenerative diseases [48]. It has been observed to cause mitochondrial dysfunction and damage to the blood-brain barrier due to cerebrovascular cell injury [49], as well as cognitive impairment, amyloid deposition [50], and α-synuclein aggregation [51]. These still need to be studied further for the development of oxidative stress in the mouse brain by *T. gondii*.

Previous studies have demonstrated the occurrence of p53-dependent apoptosis in zebrafish embryos that underwent knockdown of DJ-1 [18]. In vitro, DJ-1 interacts directly with p53 to hinder its transcriptional activity [52]. Suppression of DJ-1 resulted in elevated levels of apoptotic proteins and caspase-3 activation in cultured cells, consequently leading to an increase in the count of apoptotic cells [20]. NF-κB is a transcription factor that is tightly regulated and plays a crucial role in the regulation of apoptosis, cell proliferation, and inflammation [53]. Khalaf et al. reported that cystic fibrosis cells induce apoptosis via the NF-κB pathway [54]. Similarly, Shao et al. found that miR-146a-5p promotes IL-1β-induced chondrocyte apoptosis through the TRAF6-mediated NF-κB pathway [55]. *T. gondii* primarily parasitizes through the secretion of various parasitic proteins, including rhoptry protein (ROP), dense granules (GRA), and micronemes (MIC) [56, 57]. Among these proteins, GRA15_II_ has been demonstrated to modulate the host nuclear factor NF-κB pathway, which affects parasite growth, cytokine levels, and apoptosis through the activation of high levels of p65 translocation [32]. GRA15_II_ has also been linked to the activation of pro-apoptotic pathways, macrophage activation, and stimulation of Th1-responsive immunity [32]. The endemic Chinese type 1 (ToxoDB #9) strains found in China by our laboratory carry both GRA15_II_ and ROP16_I/III_ effectors [58]. TgCtwh3 and TgCtwh6 are the representative strains [28]. In our study, we observed that the infection of HT22 cells with TgCtwh3 tachyzoites upregulated the expression of *p*-NF-κBp65 and NF-κBp65 in the cells. Furthermore, the expression of apoptosis-related proteins, p53 and caspase-3, was also elevated, indicating that TgCtwh3 tachyzoites induce apoptosis through the NF-κB signaling pathway in HT22 cells. Additionally, we found that knockdown of DJ-1 expression in HT22 cells also led to apoptosis. Moreover, after knockdown of HT22 cells and subsequent infection with TgCtwh3 tachyzoites, the expression of NF-κBp65, *p*-NF-κBp65, p53, and caspase-3 was significantly elevated. Therefore, we suggest that in the absence of DJ-1, TgCtwh3 can induce more severe apoptosis in HT22 cells through the NF-κB signaling pathway.

Additionally, studies have demonstrated an upregulation of DJ-1 expression in the brain of an Alzheimer’s disease model [18], which is characterized by the accumulation of β-amyloid, neuronal apoptosis, and hyperphosphorylation of Tau proteins [59]. Furthermore, it has been observed that *T. gondii* RH strains activate the NF-κB signaling pathway to inhibit apoptotic gene expression after infection [60]. The discrepancy between these findings and our own may be attributed to variations in cell lines and *T. gondii* genotypes utilized in the experiments, as the prevalent RH strains in Europe and the United States possess a non-functional GRA15_II_ due to a frameshift deletion [32].

Several recent studies have demonstrated that *Toxoplasma* infections result in inflammation, which is linked to elevated levels of oxidative stressors caused by an increase in reactive oxygen species [61, 62]. Nevertheless, the host cells are safeguarded against infection-induced excess free radicals by antioxidant defenses [63]. Glutathione (GSH) is one such factor that plays a crucial role in antioxidant defense against oxidative stress [61, 63]. DJ-1 has been demonstrated to stimulate the expression of glutamate cysteine ligase, which is a crucial enzyme in the synthesis of GSH [64]. Research has shown that DJ-1 deficiency impacts central metabolism by decreasing glutamine endocytosis and serine biosynthesis [65]. Therefore, we hypothesized that knockdown of DJ-1 expression in HT22 cells reduces glutamine and serine metabolism, thereby causing oxidative damage. This is followed by TgCtwh3 tachyzoite infection, which in turn leads to increased HT22 apoptosis.

In conclusion, we found in this study that TgCtwh3 tachyzoite infection resulted in decreased DJ-1 expression, which contribute to the apoptosis observed in HT22 cells and mouse hippocampal neurons. Additionally, knockdown of DJ-1 expression in HT22 cells promoted apoptosis. Furthermore, knockdown of DJ-1 expression in HT22 cells followed by infection with TgCtwh3 tachyzoites resulted in increased apoptosis through the NF-κB signaling pathway. We assume that GRA15_II_ in TgCtwh3 activates p65 translocation and DJ-1 may play an important role in protecting neurons from apoptosis. We believe that DJ-1 is a promising gene and we can continue to investigate its association with TgCtwh3 tachyzoite infection as a target. We will also further validate our hypothesis in future work.

## Data Availability

All articles from which data was cited to support the conclusions of this manuscript are listed in the text and the reference.
